# The “Dark Side” Effects of Social Capital on Harmful Drinking among Chinese Community Residents: A Multilevel Study

**DOI:** 10.3390/ijerph15102249

**Published:** 2018-10-15

**Authors:** Xin Nie, Yongkai Zhu, Hua Fu, Junming Dai, Junling Gao

**Affiliations:** Department of Preventive Medicine and Health Education, School of Public Health, Fudan University, Shanghai 200032, China; 17211020079@fudan.edu.cn (X.N.); 16211020075@fudan.edu.cn (Y.Z.); hfu@fudan.edu.cn (H.F.); jmdai@fudan.edu.cn (J.D.)

**Keywords:** harmful drinking, neighborhood residents, dark side, social capital, multilevel modeling

## Abstract

*Background*: To determine the effects of social capital on harmful drinking (HD) among Chinese community residents using a multilevel study. *Methods*: A cross-sectional study conducted from 2017–2018. In total, 13,610 participants were randomly interviewed from 29 districts of 3 cities in China with a multi-stage sampling procedure. Social capital, including social cohesion, membership in social organizations, and frequency of social participation, were assessed using validated scales. HD was assessed using the CAGE four-item questionnaire. Multilevel models were developed to determine whether social capital was related to HD when socioeconomic and demographic covariates were controlled. *Results*: In general, the prevalence of HD was 8.18%, and more specifically, 13.77% for men and 2.74% for women. After controlling for covariates and stratifying by gender, compared to residents in the low individual-level membership of social organizations, we found that the odds ratio (OR) for HD was 1.30 with a 95% confidence interval (CI) of 1.07–1.56 among men and 1.95 (95% CI: 1.29–2.97) among women. Compared to residents in the low individual-level frequency of social participation groups, the odds ratio of HD among women was 1.58 (95% CI: 1.10–2.26). There was no association between district-level social capital and HD. *Conclusions*: A high level of social capital may promote HD among the residents of Chinese neighborhoods. Intervention to modify social capital under the Chinese drinking culture may help reduce HD.

## 1. Introduction

Harmful drinking (HD) is the most significant public health challenge because of its serious individual and collective consequences, including poor health outcomes, loss of productivity, increased mortality, association with more than 60 diseases, and increased propensity for violence and crime [1,2,3,4]. In 2016, a study indicated that adults aged 18 years and older consume approximately 3 liters (3 L) of any kind of alcohol per year (5.6 L by men and 0–3 L by women). The prevalence of HD was 9.3% on average, with 11.1% among men and 2.0% among women [5]. There were factors at the individual and societal levels that were related to alcohol consumption. The individual-level factors included age, gender, and socioeconomic status, and the societal-level factors included culture; drinking context; level of social and economic development; and alcohol production, distribution, and management [6]. However, there are a few studies focusing on the social impart of alcoholism or harmful drinking, and these studies have received more attention in recent years [7,8].

Social capital can affect health as a social determinant and studies have shown that social capital was related to some health outcomes, such as physical health, mental health, violent crime, and even mortality [4,8,9,10,11,12,13]. Social capital, including structural (networking, connections, and citizen participation) and cognitive components (trust, social cohesion, and social support), enables people to take collective action, cooperate, and participate in social organizations [14]. Previous studies showed that social capital can impact health at individual and collective levels, which indicated that social capital should be assessed at both levels [9,15]. At the collective level, social capital is commonly measured by summarizing individual perceptions of social capital [15].

Although plenty of studies showed that social capital is negatively associated to HD [9,16,17,18,19], some studies found that social capital is a harmful social determinant; for example, it will cause pressure on individuals who are obsessed with social networks such that harmful social cohesion and norms are maintained [20]. Furthermore, it was found that social participation can lead to occasional heavy drinking [8]. Abdu’s research showed that some aspects of social capital, including social participation and social trust, were positively correlated with the risk of single drinking and found that social capital provides little protection [21]. Drinking culture in Denmark is quite similar to the drinking culture in China [22]. Family members, friends, and colleagues are likely to drink to intoxication and tell funny and interesting stories in order to show good friendship and respect to each other [23]. Chuang found that for both men and women, membership in social organizations is positively correlated with HD [24].

Our previous study found that workplace social capital was a protective factor for HD [25], but it mainly focused on an occupational population. Considering the previous findings about the mixed effects of the structural and cognitive components of social capital on HD, our study’s aim was to examine the relationship between some indicators of social capital at individual- and district-level, including social cohesion and membership in social organizations with the frequency and prevalence of HD. Given these results under the drinking culture in China, and considering the multilevel effect of social capital, we hypothesize that three dimensions of social capital could be positively associated with HD.

## 2. Materials and Methods

### 2.1. Design and Participants

This study was conducted in Shanghai, Zhengzhou, and Xinzheng in Henan province, China from June 2017 to March 2018. Neighborhood residents from 29 districts were randomly selected using multi-staged sampling. All 16 districts in Shanghai, all 7 districts in Zhengzhou, and 6 districts randomly selected from Xinzheng were selected as clusters, then 5% of the population aged 15 to 75 years old were randomly selected from each district. Data was collected using a self-administered “health city needs assessment questionnaire” This study has been approved by the Institutional Review Board of Fudan University (Ethic Approval Code: IRB#2018-03-0666).

### 2.2. Measurement

#### 2.2.1. Harmful Drinking

The CAGE (focusing on Cutting down, Annoyance by criticism, Guilty feeling, and Eye-openers) four-item questionnaire was used to measure HD (see Supplementary Materials Table S1). The four items were summed using scoring from 0 to 4 [25]. A higher score indicates an alcohol-related problem and a total of 2 or more is considered to signify the prevalence of HD, which is clinically significant [26]. The Cronbach’s alpha was 0.65.

#### 2.2.2. Social Capital

Because there was no uniform social capital concept and measure, we used social cohesion to measure the cognitive component and membership of social organizations and frequency of social participation to measure the structural component based on previous studies [27].

(1) Social cohesion: It was assessed using the Chinese version of the Neighborhood Scales (see Supplementary Table S2) [13,28]. Each of the four items ranged from 1 to 5 (from strongly disagree to strongly agree), and the total score was added by the score of the four items. The Cronbach’s alpha was 0.91.

(2) Membership in social organizations: We assessed it by asking participants whether they were a member of the following six social organizations in their respective district (see Supplementary Materials Table S3). Participants responded each item to “yes” (scoring 1) or “no” (scoring 0), and a total score was added to give the score of the six items [27]. The Cronbach’s alpha was 0.79.

(3) Frequency of social participation: We assessed this by asking participants how often they participated in eight different activities (see Supplementary Materials Table S4) [27]. Each social participation ranged from 1 to 5 (1 = never, 2 = several times a year, 3 = several times a month, 4 = once a week, and 5 = two or more times a week), and a total score was added to give the score of the eight items. The Cronbach’s alpha was 0.86.

To analyze the three dimensions of social capital, we assessed them at the individual and aggregate levels. Both levels of social capital scores were dichotomized into high versus low level using the median [9,13].

#### 2.2.3. Covariates

Base on a literature review [29,30,31,32], the following covariates were included the current study.

(1) Smoking status

Smoking status was measured using two questions: (1) have you smoked more than 100 cigarettes in your entire life? (2) Have you smoked, even one puff, in the past 30 days? Current smokers responded “yes” to both questions; otherwise, they were regarded as nonsmokers [11].

(2) Mental health

WHO-5 Wellbeing Index was used to assess mental health (see Supplementary Materials Table S5) [33]. Each of the five items were scored from 5 (all of the time) to 0 (none of the time). A total score above 13 meant good mental health, while below 13 indicated poor mental health. The Cronbach’s alpha was 0.94.

(3) Self-rated health 

Self-rated health (SRH) is a commonly-used health indicator for the association between social capital and health [12]. In our questionnaire, it was a five-point scale, in which 1 = extremely good, 2 = very good, 3 = good, 4 = fair, and 5 = poor.

#### 2.3.4. Other Covariates

Several sociodemographic and socioeconomic factors were included as covariates: gender, age categorized in 10-year increments (ranging from ≤29 to ≥70 years old), occupation status (employed vs unemployed, including students, unemployed, and retirement), marital status (married vs other, including unmarried, divorced, and widowed), region of origin (native vs immigrant). In addition, participants came from three cities: Shanghai, Zhengzhou, and Xinzheng.

### 2.3. Statistical Analyses

At the first step, a χ^2^ test was used for univariate analysis, and data from this study had a multilevel structure comprised of neighborhood residents (at level 1) nested within districts (at level 2). The analysis proceeded as follows [34]: (1) Empty Model: we examined the district factor with HD without any variables; (2) Model 1: we examined the individual-level of social capital and HD; (3) Model 2: we examined the district-level of social capital and HD; (4) Model 3: we examined the relationship of both individual- and district-level of social capital and HD. For Models 1–3, we controlled all the covariates: smoking status, mental health, self-rated health, gender, age, occupation status, marital status and region of origin, and used −2 log likelihood (−2LL) and Akaike information criterion (AIC) to assess the goodness-of-fit of each model [13], and the significant *p*-value was set at <0.05 (two tailed). The STATA version 13.0 program (StataCorp LP., College Station, TX, USA) was used for the whole analysis [13,34].

## 3. Results

### 3.1. Descriptive Results

In total, 13,610 residents (48.43% men and 51.57% women) with a mean ± standard deviation (SD) age of 46.89 ± 15.01 years responded. Of these, 30.96% had enrolled in the university, 64.95% were employed, 86.64% were married, and 81.22% were native. In general, the prevalence of HD was 8.18%, more specifically, 13.77% for men and 2.74% for women.

Descriptive results of sociodemographic characteristics and the prevalence of HD are given in Table 1. The prevalence of HD was different in educational levels: those with lower education (elementary school) had a higher prevalence, and those with higher education (university or higher) had a lower prevalence. The prevalence was higher among employed men (14.98%) than among unemployed men (10.72%), but there was no difference among women. The prevalence was higher among married men (14.12%) than among other conditions (10.20%), but the prevalence showed no statistically significant difference among women. HD also significantly varied by city, with the prevalence among participants from Zhengzhou being highest and the prevalence among participants from Shanghai being lowest among both men and women. There were no significant differences in HD distributions among regions of origin and self-rated health among either men or women. The rate of HD was much higher (23.23% for men and 15.66% for women) among those who were currently smoking than among those who never smoked or were former smokers (8.02% for men and 2.35% for women). The HD prevalence showed no difference between high and low individual-level social cohesion among either men or women. Among men, the prevalence of HD ascended with high individual-level membership in social organizations and frequency of social participation but did not ascend with individual-level social cohesion; among women, the prevalence of HD ascended with high individual-level social cohesion and membership in social organizations but did not ascend with individual-level frequency of social participation.

### 3.2. Multilevel Logistic Regression of the Association between Social Capital and HD

Since the prevalence of HD was much higher among men, we modeled the relationships between social capital and HD among men and women. The multilevel logistic model results are shown in Table 2 for men and Table 3 for women. The empty (null) model revealed that there was a significant difference in the prevalence of HD across districts among men (χ^2^ = 422.26, *p* < 0.001) and women (χ^2^ = 201.49, *p* < 0.001). The intraclass correlation coefficients (ICC) were 0.169 for men and 0.178 for women, indicating that 16.9% (men) and 17.8% (women) of the variation was explained by a random effect by the different districts.

The results of the model showed that the adjusted OR of social capital and HD were greater among those who were employed (men’s OR: 1.43, 95% CI: 1.14–1.80; women’s OR: 1.76, 95% CI: 1.18–2.63), current smokers (men’s OR: 3.36, 95% CI: 2.87–3.93; women’s OR: 7.66, 95% CI: 4.81–12.19), and residents with good mental health (men’s OR: 1.39, 95% CI: 1.08–1.78; women’s OR: 1.85, 95% CI: 1.03–3.31). Additionally, among women, the prevalence of HD was lower at higher education levels, where university or higher levels of education had the lowest odds ratio of HD (OR: 0.36, 95% CI: 0.21–0.63). Of particular interest, associations between three dimensions of individual-level social capital and the prevalence of HD were different after controlling for covariates and stratifying by gender. At the individual-level, the adjusted OR were greater among those who had a higher level of membership in social organizations (men’s OR: 1.52, 95% CI: 1.27–1.82; women’s OR: 2.08, 95% CI: 1.40–3.10), and the adjusted OR were greater among women who had a higher level of social cohesion (OR: 1.45, 95% CI: 1.03–2.04).

In model 2, we found that none of three dimensions of district-level social capital were related to HD, but some individual-level covariates were significantly associated with HD: current smokers (men’s OR: 3.28, 95% CI: 2.79–3.86; women’s OR: 8.71, 95% CI: 5.38–14.11) and residents with higher mental health scores (men’s OR: 1.64, 95% CI: 1.27–2.11; women’s OR: 1.96, 95% CI: 1.09–3.54). Among women, the prevalence of HD was lower with higher educational levels and lowest with a university or higher educational level (OR: 0.45, 95% CI: 0.25–0.81). The adjusted OR of HD were lower among men that were local residents (OR: 0.78, 95% CI: 0.63–0.97).

In model 3, after controlling for district-level social capital, the results of model 3 indicated that the associations between the covariates and the HD were comparable between model 1 and 2, and among women, mental health status was not significantly associated with HD. Furthermore, the associations between the three dimensions of social capital and HD were comparable between model 1 and 2. Neither the individual-level frequency nor district-level frequency of social participation was associated with HD.

## 4. Discussions

To our best knowledge, it is the first time that the “dark side” effects of social capital impacting HD were found in the Chinese context. We found that a high membership rate in social organizations was associated with a high prevalence of HD among men, and high social cohesion and membership in social organizations were associated with a high prevalence of HD among women; the results were consistent with those of previous studies [8,21,24,35]. Seid et al. found that membership in voluntary organizations among women was positively associated with harmful drinking in the Danish general population [21]. Measured by the CAGE questionnaire, Murphy et al. also found that community social participation was positively related with HD. Chuang also indicated that membership in social organizations was positively associated with harmful drinking in both genders [24].

There are several plausible explanations for social capital positively associated with HD, but there are differences between men and women. First, for both men and women, high-level membership in social organizations was positively associated with a high prevalence of HD. This is similar to the drinking culture in Denmark [8,23], and alcohol is an important part of Chinese culture. Drinking, as a part of a business meeting, is very common in China, especially for men, and will help to form good business relationships between supervisors and colleagues [36]. As the Song Dynasty poet, Ouyang Xiu said, “one thousand cups is not enough for a bosom friend” [37], and in such a culture, enhancing sociability may result in HD. Second, unlike other studies, we found that social cohesion was positively associated with HD only among women. There are “double standards” that are applied to drinking between men and women. Specifically, women are much more likely to be sensitive to societal disapproval [36]. Additionally, women were more likely to have more devoted friends, which often leads to a trusting relationship [24]. Therefore, Chinese women may drink excessively during social intercourse with close friends.

The different measurements of HD and social capital may cause the inconsistency of our findings with other “negative relation” studies [9]. Gao et al. measured drinking using the Chinese version of Alcohol Use Disorders Identification Test (AUDIT) assess hazardous drinking, while we used the CAGE questionnaire. Twenty-five years after the CAGE questionnaire was published, numerous studies have confirmed that the questionnaire is a good and quick primary indicator that further investigation is needed [38]. The HD indicator was firstly developed to identify alcohol problems in hospitalized patients and remains one of the most widely-used screening tools [26]. Therefore, we chose the CAGE questionnaire as our measurement for HD. Furthermore, previous research mainly focused on cognitive workplace social capital, while the current study measured both cognitive and structural components of social capital in neighborhoods.

Nevertheless, no relationships between district-level social capital and HD were found in the study. In China, social capital mainly exists within the family or other social organizations, which means that people trust those belonging to the same group [39], so the impact of social capital on HD may be diluted and less relevant when we aggregate individual-level social capital into the district-level. Our study found that neither the individual-level frequency nor district-level social participation frequency was associated with HD. A previous study in Taiwan [24] indicated that social participation was positively associated with drinking. The inconsistency of this current finding with prior studies may be because of the difference in the way harmful drinking was assessed. The CAGE four-item questionnaire focuses on the consequences of alcohol consumption [26], while other studies measured drinking behavior by asking respondents whether they drink frequently. Therefore, the CAGE may underestimate the rate of HD. Further studies should be focused on the social participation frequency and HD. 

In the multilevel models, other covariates were associated with HD. For example, native men had a lower rate of HD than non-local men who have a rural “Hukou” (a registration as a permanent rural resident). These results were similar to those of a previous study indicating that rural–urban migrants were more likely to engage in HD than urban residents due to the greater number of work-related stressors they experience [40]. Moreover, women with higher education levels had a lower rate of HD because the higher the education level was, the higher the acceptance of alcohol education was, where women with higher education may discourage harmful drinking [41]. In our study, we found that residents who had a good mental health status were more likely to engage in HD. However, a previous study indicated that harmful drinking is associated with higher risks of mental health [29], which is quite different from our study. The positive relation between good mental health and the high rates of HD should be studied further.

There are some limitations in our study. First, the data from this cross-sectional study prohibited us from drawing conclusions about causality. In other words, we cannot tell social capital caused HD or vice versa. Further longitudinal studies, such as cohort studies or nested case control studies, that investigate the relationship between social capital and HD from various industries is imperative. Additionally, the data were from a self-administered questionnaire, so the possibility of a lack of attention or non-professional investigators should be considered [35]. Second, there is a tendency for residents to underreport their harmful drinking status due to a suspicion that other people could gain the answers that are considered private [8]. Third, our study might have missed those with severe drinking problems (e.g., intoxicated, homeless), thereby producing somewhat conservative estimates of the relationship between social capital and HD. Finally, we estimated social capital using social cohesion, membership in social organizations, and frequency of social participation, and we need to add other aspects of social capital, such as social trust, in further studies. Compared with the Alcohol Use Disorders Identification Test (AUDIT) questionnaires, The CAGE is easier to understand but the CAGE may underestimate the HD prevalence [42], which may interfere with the association between social capital and HD.

## 5. Conclusions

Our study found positive associations between three dimensions of social capital and HD, and for the first time, we revealed the “dark side” of social capital impacting HD in China and the Chinese drinking culture that may influence drinking behaviors. After stratification by gender, we discovered that individual- and district-level social cohesion were positively related to HD only among women. In contrast, there was no association between district-level social capital and HD. In light of these findings, we should administer some longitudinal studies to examine the relationship between additional aspects of social capital and HD. Interventions regarding creating a healthy drinking environment and a rational usage of social capital may contribute to reduce the rate of harmful drinking.

## Figures and Tables

**Table 1:** Comparisons of the rates of HD among demographic characteristics using a univariate analysis.

Characteristic	Men	Harmful Drinking *n* (%)	*p*	Women	Harmful Drinking *n* (%)	*p*
	Frequency (%)			Frequency (%)		
All	6375 (100)	878 (13.77)		6787 (100)	186 (2.74)	
Age						
≤29	971 (15.23)	109 (11.23)	<0.001	937 (13.81)	25 (2.67)	0.622
30–39	1220 (19.14)	170 (13.93)		1209 (17.81)	28 (2.32)	
40–49	1024 (16.06)	176 (17.19)		1150 (16.94)	31 (2.70)	
50–59	1102 (17.29)	180 (16.33)		1171 (17.25)	40 (3.42)	
60–69	992 (15.56)	132 (13.31)		1269 (18.70)	37 (2.92)	
≥70	1066 (16.72)	111 (10.41)		1051 (15.49)	25 (2.38)	
Educational level						
Elementary school	749 (11.83)	130 (17.36)	<0.001	749 (11.06)	36 (4.81)	0.001
Junior high school	1934 (30.54)	298 (15.41)		1957 (28.90)	57 (2.91)	
Senior high school	1729 (27.31)	230 (13.30)		1925 (28.43)	49 (2.55)	
University	1920 (30.32)	218 (11.35)		2140 (31.61)	44 (2.06)	
Occupation						
Employed	4578 (72.74)	686 (14.98)	<0.001	3809 (57.00)	113 (2.97)	0.159
Unemployed	1716 (27.26)	184 (10.72)		2874 (43.00)	69 (2.40)	
Marital status						
Married	5516 (87.33)	779 (14.12)	0.005	5807 (86.17)	156 (2.69)	0.580
Other	800 (12.67)	84 (10.20)		932 (13.83)	28 (3.00)	
City						
Shanghai	3594 (56.38)	386 (10.74)	<0.001	4339 (63.93)	101 (2.33)	0.008
Zhengzhou	1454 (22.81)	267 (18.36)		1648 (24.28)	52 (3.16)	
Xinzheng	1327 (20.82)	225 (16.96)		800 (11.79)	33 (4.13)	
Region of Origin						
Native	4805 (79.63)	658 (13.69)	0.280	5314 (82.50)	153 (2.88)	0.117
Immigrant	1229 (20.37)	183 (14.89)		1127 (17.50)	23 (2.04)	
Self-rated health						
Excellent	884 (13.98)	123 (13.91)	0.093	657 (9.74)	24 (3.65)	0.198
Fine	1991 (31.48)	248 (12.46)		1879 (27.87)	42 (2.24)	
Well	2062 (32.60)	289 (14.02)		2206 (32.72)	58 (2.63)	
General/Low	1388 (21.94)	215 (15.49)		2001 (29.68)	61 (3.05)	
Smoking status						
Current	2411 (37.82)	560 (23.23)	<0.001	198 (2.92)	31 (15.66)	<0.001
Never/former	3964 (62.18)	318 (8.02)		6589 (97.08)	155 (2.35)	
Mental health						
Good	5541 (86.92)	773 (13.95)	0.288	5973 (88.01)	168 (2.81)	0.324
Poor	834 (13.08)	105 (12.59)		814 (11.99)	18 (2.21)	
Individual-level social cohesion						
High	3507 (55.01)	503 (14.34)	0.144	3876 (57.11)	120 (3.10)	0.038
Low	2868 (44.99)	375 (13.08)		2911 (42.89)	66 (2.27)	
Individual-level membership in social organizations						
High	3647 (57.21)	574 (15.74)	<0.001	4178 (61.56)	141 (3.37)	<0.001
Low	2728 (42.79)	304 (11.14)		2609 (38.44)	45 (1.72)	
Individual-level frequency of social participation						
High	3209 (50.34)	470 (14.65)	0.042	3711 (54.68)	103 (2.78)	0.846
Low	3166 (49.66)	408 (12.89)		3076 (45.32)	83 (2.70)	

**Table 2:** The odds ratios and 95% confidence intervals for HD associated with individual and district-level social capital among men.

Men	Empty Model	Model 1	Model 2	Model 3
	OR (95% CI)	OR (95% CI)	OR (95% CI)	OR (95% CI)
Fixed effects				
Age (year)				
≤29		1	1	1
30–39		1.18 (0.88–1.59)	1.34 (0.99–1.82)	1.34 (0.99–1.83)
40–49		1.45 (1.07–1.97)	1.71 (1.24–2.36)	1.71 (1.24–2.36)
50–59		1.34 (0.98–1.83)	1.81 (1.30–2.52)	1.78 (1.30–2.49)
60–69		1.20 (0.85–1.71)	1.85 (1.28–2.69)	1.75 (1.21–2.55)
≥70		0.90 (0.65–1.26)	1.12 (0.79–1.57)	1.10 (0.78–1.55)
Educational level				
Elementary school		1	1	1
Junior high school		0.83 (0.65–1.07)	0.95 (0.74–1.24)	0.95 (0.73–1.23)
Senior high school		0.74 (0.57–0.96)	0.87 (0.66–1.15)	0.85 (0.64–1.12)
University		0.64 (0.49–0.85)	0.78 (0.58–1.05)	0.75 (0.56–1.00)
Employed (vs. Unemployed)		1.43 (1.14–1.80)	1.16 (0.92–1.47)	1.17 (0.92–1.47)
Married (vs. Other)		1.18 (0.89–1.56)	1.13 (0.84–1.51)	1.13 (0.84–1.51)
Native (vs. Immigrant)		0.89 (0.73–1.09)	0.78 (0.63–0.97)	0.74 (0.59–0.92)
Self-evaluated health condition				
Excellent		1	1	1
Fine		0.81 (0.63–1.05)	0.92 (0.71–1.19)	0.92 (0.70–1.19)
Well		0.94 (0.73–1.21)	1.05 (0.81–1.36)	1.05 (0.81–1.37)
General/Low		1.17 (0.89–1.54)	1.23 (0.92–1.63)	1.25 (0.94–1.66)
Current smoking (vs. Never/former)		3.36 (2.87–3.93)	3.28 (2.79–3.86)	3.34 (2.81–3.93)
Good mental health (vs. Poor)		1.39 (1.08–1.78)	1.64 (1.27–2.11)	1.58 (1.22–2.05)
High Individual-level social cohesion (vs. Low)		0.99 (0.84–1.16)		1.04 (0.88–1.23)
High Individual-level membership in social organizations (vs. Low)		1.52 (1.27–1.82)		1.30 (1.07–1.56)
High Individual-level frequency of social participation (vs. Low)		1.16 (0.97–1.37)		1.08 (0.91–1.29)
High District-level social cohesion (vs. Low)			1.09 (0.59–2.02)	1.06 (0.57–1.95)
High District-level membership in social organizations (vs. Low)			1.80 (0.85–3.77)	1.64 (0.78–3.41)
High District-level frequency of social participation (vs. Low)			1.17 (0.58–2.36)	1.16 (0.58–2.32)
Model fit				
−2LL	4833.64	4431.64	4225.11	4212.87
AIC	4837.64	4455.64	4267.11	4260.87

**Table 3:** The odds ratios and 95% confidence intervals for HD associated with individual and district-level social capital among women.

Women	Empty Model	Model 1	Model 2	Model 3
	OR (95% CI)	OR (95% CI)	OR (95% CI)	OR (95% CI)
Fixed effects				
Age (year)				
≤29		1	1	1
30–39		0.91 (0.49–1.68)	1.02 (0.55–1.92)	0.98 (0.52–1.85)
40–49		0.87 (0.46–1.64)	1.02 (0.53–1.99)	0.93 (0.48–1.82)
50–59		1.32 (0.69–2.56)	1.69 (0.85–3.38)	1.50 (0.75–3.01)
60–69		1.06 (0.53–2.11)	1.53 (0.74–3.16)	1.21 (0.58–2.54)
≥70		0.61 (0.31–1.22)	0.80 (0.39–1.64)	0.71 (0.35–1.46)
Educational level				
Elementary school		1	1	1
Junior high school		0.52 (0.32–0.82)	0.53 (0.32–0.87)	0.55 (0.33–0.90)
Senior high school		0.47 (0.29–0.76)	0.53 (0.31–0.89)	0.51 (0.30–0.87)
University		0.36 (0.21–0.63)	0.45 (0.25–0.81)	0.43 (0.24–0.79)
Employed (vs. Unemployed)		1.76 (1.18–2.63)	1.18 (0.77–1.79)	1.24 (0.81–1.89)
Married (vs. Other)		0.89 (0.55–1.45)	0.89 (0.54–1.48)	0.89 (0.54–1.48)
Native (vs. Other)		1.37 (0.85-2.21)	1.41 (0.85–2.33)	1.28 (0.77–2.13)
Self-evaluated health condition				
Excellent		1	1	1
Fine		0.59 (0.35–1.00)	0.57 (0.33–0.99)	0.56 (0.32–0.96)
Well		0.64 (0.38–1.09)	0.62 (0.36–1.06)	0.64 (0.37–1.10)
General/Low		0.86 (0.50–1.48)	0.69 (0.39–1.21)	0.79 (0.45–1.38)
Current smoking (vs. Never/former)		7.66 (4.81–12.19)	8.71 (5.38–14.11)	9.28 (5.65–15.23)
Good mental health (vs. Poor)		1.85 (1.03–3.31)	1.96 (1.09–3.54)	1.77 (0.97–3.21)
High Individual-level social cohesion (vs. Low)		1.45 (1.03–2.04)		1.58 (1.10–2.26)
High Individual-level membership in social organizations (vs. Low)		2.08 (1.40–3.10)		1.95 (1.29–2.97)
High Individual-level frequency of social participation (vs. Low)		0.81 (0.57–1.15)		0.92 (0.65–1.32)
District-level High social cohesion (vs. Low)			0.91 (0.37–2.21)	0.79 (0.32–1.98)
High District-level membership in social organizations (vs. Low)			1.29 (0.44–3.73)	1.13 (0.41–3.15)
High District l-level frequency of social participation (vs. Low)			1.15 (0.42–3.13)	1.13 (0.41–3.15)
Model fit				
−2LL	1628.10	1462.50	1410.26	1389.33
AIC	1632.10	1502.50	1452.26	1437.33

## Supplementary Materials

The following are available online at http://www.mdpi.com/1660-4601/15/10/2249/s1, Table S1: CAGE-4 item questionnaire was used to assess harmful drinking, Table S2: Neighborhood Scales used to assess social cohesion, Table S3: Membership in social organizations used in the study, Table S4: Frequency of social participation used in the study, Table S5: WHO-5 Well-Being Index was used to assess mental health.

## References

[1] Bouchery E.E., Harwood H.J., Sacks J.J., Simon C.J., Brewer R.D. (2011). Economic costs of excessive alcohol consumption in the US, 2006. Am. J. Prev. Med..

[2] Rehm J., Mathers C., Popova S., Thavorncharoensap M., Teerawattananon Y., Patra J. (2009). Global burden of disease and injury and economic cost attributable to alcohol use and alcohol-use disorders. Lancet.

[3] Gutjahr E., Gmel G., Rehm J. (2001). Relation between average alcohol consumption and disease: An overview. Eur. Addict. Res..

[4] Rowland B.C., Wolfenden L., Gillham K., Kingsland M., Richardson B., Wiggers J. (2015). Is alcohol and community sport a good mix? Alcohol management, consumption and social capital in community sports clubs. Aust. N. Z. J. Public Health.

[5] Gu J. (2016). Interpretation of China national nutrition and chronic disease status survey(2015). Acta Nutr. Sin..

[6] WHO Alcohol. http://www.who.int/en/news-room/fact-sheets/detail/alcohol.

[7] Schmidt L.A., Mäkelä P., Rehm J., Room R., Blas E., Kurup A.S. (2010). Alcohol: Equity and social determinants. Equity, Social Determinants and Public Health Programmes.

[8] Murphy A., Roberts B., Kenward M.G., De Stavola B.L., Stickley A., McKee M. (2014). Using multi-level data to estimate the effect of social capital on hazardous alcohol consumption in the former Soviet Union. Eur. J. Public Health.

[9] Gao J., Weaver S.R., Fua H., Pan Z. (2014). Does workplace social capital associate with hazardous drinking among Chinese rural-urban migrant workers?. PLoS ONE.

[10] Gao J., Weaver S.R., Dai J., Jia Y., Liu X., Jin K., Fu H. (2014). Workplace social capital and mental health among Chinese employees: A multi-level, cross-sectional study. PLoS ONE.

[11] Gao J., Nehl E.J., Fu H., Jia Y., Liu X., Zheng P. (2013). Workplace social capital and smoking among Chinese male employees: A multi-level, cross-sectional study. Prev. Med..

[12] Meng T., Chen H. (2014). A multilevel analysis of social capital and self-rated health: Evidence from China. Health Place.

[13] Gao J., Fu H., Li J., Jia Y. (2015). Association between social and built environments and leisure-time physical activity among Chinese older adults—A multilevel analysis. BMC Public Health.

[14] Islam M.K., Merlo J., Kawachi I., Lindström M., Gerdtham U.-G. (2006). Social capital and health: Does egalitarianism matter? A literature review. Int. J. Equity Health.

[15] Murayama H., Fujiwara Y., Kawachi I. (2012). Social capital and health: A review of prospective multilevel studies. J. Epidemiol..

[16] Takakura M. (2011). Does social trust at school affect students’ smoking and drinking behavior in Japan?. Soc. Sci. Med..

[17] Larm P., Åslund C., Starrin B., Nilsson K. (2016). How are social capital and sense of coherence associated with hazardous alcohol use? Findings from a large population-based Swedish sample of adults. Scand. J. Public Health.

[18] Zhai H., Yang Y., Sui H., Wang W., Chen L., Qiu X., Yang X., Qiao Z., Wang L., Zhu X. (2015). Self-esteem and problematic drinking in China: A mediated model. PLoS ONE.

[19] Åslund C., Nilsson K.W. (2013). Social capital in relation to alcohol consumption, smoking, and illicit drug use among adolescents: A cross-sectional study in Sweden. Int. J. Equity Health.

[20] Portes A. (1998). Social capital: Its origins and applications in modern sociology. Annu. Rev. Sociol..

[21] Seid A.K., Hesse M., Bloomfield K. (2016). ‘Make it another for me and my mates’: Does social capital encourage risky drinking among the Danish general population?. Scand. J. Public Health.

[22] Järvinen M. (2003). Drinking rituals and drinking problems in a wet culture. Addict. Res. Theory.

[23] Tutenges S., Sandberg S. (2013). Intoxicating stories: The characteristics, contexts and implications of drinking stories among Danish youth. Int. J. Drug Policy.

[24] Chuang Y.C., Chuang K.Y. (2008). Gender differences in relationships between social capital and individual smoking and drinking behavior in Taiwan. Soc. Sci. Med..

[25] Chen Y.T., Ibragimov U., Nehl E.J., Zheng T., He N., Wong F.Y. (2016). Validity of the CAGE questionnaire for men who have sex with men (MSM) in China. Drug Alcohol Depend..

[26] O’shea R.S., Dasarathy S., McCullough A.J. (2010). Alcoholic liver disease. Hepatology.

[27] Takeuchi K., Aida J., Kondo K., Osaka K. (2013). Social participation and dental health status among older Japanese adults: A population-based cross-sectional study. PLoS ONE.

[28] Mujahid M.S., Diez Roux A.V., Morenoff J.D., Raghunathan T. (2007). Assessing the measurement properties of neighborhood scales: From psychometrics to ecometrics. Am. J. Epidemiol..

[29] Huang R., Ho S.Y., Wang M.P., Lo W.S., Lam T.H. (2016). Reported alcohol drinking and mental health problems in Hong Kong Chinese adolescents. Drug Alcohol Depend..

[30] Lee M.R., Chassin L., MacKinnon D.P. (2015). Role transitions and young adult maturing out of heavy drinking: Evidence for larger effects of marriage among more severe premarriage problem drinkers. Alcohol. Clin. Exp. Res..

[31] Woolard R., Liu J., Parsa M., Merriman G., Tarwater P., Alba I., Villalobos S., Ramos R., Bernstein J., Bernstein E. (2015). Smoking is associated with increased risk of binge drinking in a young adult hispanic population at the US-Mexico border. Substain. Abus..

[32] Moriconi P.A., Nadeau L. (2015). A cross-sectional study of self-rated health among older adults: Association with drinking profiles and other determinants of health. Curr. Gerontol. Geriatr. Res..

[33] WHO Collaborating Centre in Mental Health Chinese version of the WHO Five Well-Being Index. http://www.who-5.org.

[34] Wang J.H., Xie H.Y., Fisher J.H. (2008). Multilevel Models: Methods and Applications.

[35] Martins J.G., de Paiva H.N., Paiva P.C.P., Ferreira R.C., Pordeus I.A., Zarzar P.M., Kawachi I. (2017). New evidence about the “dark side” of social cohesion in promoting binge drinking among adolescents. PLoS ONE.

[36] Cochrane J., Chen H.H., Conigrave K.M., Hao W. (2003). Alcohol use in China. Alcohol Alcohol..

[37] Shi X. (2011). A brief of ancient Chinese literati and wine. QUN WEN TIAN DI.

[38] O’Brien C.P. (2008). The cage questionnaire for detection of alcoholism. JAMA.

[39] Allik J., Realo A. (2004). Individualism-collectivism and social capital. J. Cross-Cult. Psychol..

[40] Mou J., Griffiths S.M., Fong H., Dawes M.G. (2013). Health of China’s rural–urban migrants and their families: A review of literature from 2000 to 2012. Br. Med. Bull..

[41] Johnson W., Kyvik K.O., Mortensen E.L., Skytthe A., Batty G.D., Deary I.J. (2011). Does education confer a culture of healthy behavior? Smoking and drinking patterns in Danish twins. Am. J. Epidemiol..

[42] McCusker M.T., Basquille J., Khwaja M., Murray-Lyon I.M., Catalan J. (2002). Hazardous and harmful drinking: A comparison of the AUDIT and CAGE screening questionnaires. QJM An Int. J. Med..

