# Supplementary material for: The “Dark Side” Effects of Social Capital on Harmful Drinking among Chinese Community Residents: A Multilevel Study
Source: Int J Environ Res Public Health. 2018 Oct 15;15(10):2249. doi: 10.3390/ijerph15102249 (PMC6210951; doi:10.3390/ijerph15102249)
Supplement: Supplementary file 1 [file ijerph-15-02249-s001.pdf]

Supplementary Table 1 CAGE-4 item questionnaire was used to assess harmful drinking.

		Answer	
1.	Have you ever felt the need to cut down on your drinking?	Yes	No
2.	Have you ever been annoyed by others criticizing your drinking	Yes	No
3.	Have you ever felt guilty about your drinking?	Yes	No
4.	Have you ever felt guilty about your drinking?	Yes	No

Supplementary Table 2 Neighborhood Scales used to assess social cohesion

Question: 'How satisfied are you with…?'				*Rating*		
1.	People around here are willing to help their neighbors	5	4	3	2	1
2.	People in my neighborhood generally get along with each other	5	4	3	2	1
3.	People in my neighborhood can be trusted	5	4	3	2	1
4.	People in my neighborhood share the same values	5	4	3	2	1

1=strongly agree, 2=agree, 3=neutral (neither agree nor disagree), 4=disagree, 5=strongly disagree

Supplementary Table 3 Membership in social organizations used in the study

Question: 'Do you participate in the following organizations?'		*Answer*	
1.	Political organizations or associations	Yes	No
2.	Industrial or professional groups	Yes	No
3.	Volunteer groups	Yes	No
4.	Senior citizens' clubs	Yes	No
5.	Religious groups or associations	Yes	No
6.	Neighborhood community associations	Yes	No

Supplementary Table4 Frequency of social participation used in the study

Question: 'How satisfied are you with…?'	*Rating*				
1. Visiting family or friends	5	4	3	2	1
2. Recreational activities involving other people	5	4	3	2	1
3. Physical and cultural activities in the neighborhood	5	4	3	2	1
4. Attending a series of lectures in the neighborhood	5	4	3	2	1
5. Self-management group, mutual-help group	5	4	3	2	1
6. Volunteer or charity work	5	4	3	2	1
7. Activities in political organizations or associations	5	4	3	2	1
8. Dining out or shopping with other people	5	4	3	2	1

1=never, 2=several times per year, 3=several times per month, 4=once per week, and 5=two or more times per week

Supplementary Table 5 WHO-5 Well-Being Index was used to assess mental health

Question: 'How did you feel at last four weeks?'	*Rating*					
1. I have felt cheerful and in good spirits	5	4	3	2	1	0
2. I have felt calm and relaxed	5	4	3	2	1	0
3. I have felt active and vigorous	5	4	3	2	1	0
4. I woke up feeling fresh and rested	5	4	3	2	1	0
5. My daily life has been filled with things that interest me	5	4	3	2	1	0

0= at no time, 1= some of the time, 2= less than half of the time, 3= more than half of the time, 4= most of the time, 5= all of the time.
